# Pathways to Kindergarten Readiness: The Roles of Second Step Early Learning Curriculum and Social Emotional, Executive Functioning, Preschool Academic and Task Behavior Skills

**DOI:** 10.3389/fpsyg.2018.01886

**Published:** 2018-10-04

**Authors:** Melodie Wenz-Gross, Yeonsoo Yoo, Carole C. Upshur, Anthony J. Gambino

**Affiliations:** ^1^Department of Family Medicine and Community Health, University of Massachusetts Medical School, Worcester, MA, United States; ^2^Department of Psychiatry, University of Massachusetts Medical School, Worcester, MA, United States; ^3^Department of Quantitative Health Sciences, University of Massachusetts Medical School, Worcester, MA, United States; ^4^Department of Educational Psychology, University of Connecticut, Storrs, CT, United States

**Keywords:** executive functioning, social-emotional skills, preschool, kindergarten readiness, low-income, SEM, mediation, Second Step Early Learning Curriculum

## Abstract

Efforts to improve the achievement gap between low-income children and their more affluent peers has led to the development of classroom interventions and curricula to increase executive functioning (EF) and social-emotional skills (SE), thought to be foundational for learning. The Second Step Early Learning (SSEL) curriculum is a commercially available curriculum designed to improve school readiness by building EF and SE skills. However, although widely used, it has not been widely studied. Modeling SSEL’s underlying theory of change, structural equation modeling (SEM) was used to longitudinally examine the effects of the curriculum on low-income preschool children’s kindergarten school readiness through the hypothesized mediating role of EF and SE skills in improving pre-academic skills and task behavior in preschool. In a cluster randomized trial, 972 children attending 63 preschool classrooms within 13 low-income Head Start or community preschools were individually tested at the beginning (T1) and end of preschool (T2, *n* = 836) and followed into kindergarten. Children’s average age at T1 was 53 months, with 51% male, 42% Anglo-American, 26% African–American, and 40% Hispanic-American. Children’s EF, social skills, pre-literacy/language, and pre-math skills were assessed by trained child assessors blind to study conditions at T1 and T2. Assessors also rated children’s task behavior in the testing situation at T1 and T2. School records of children’s kindergarten screening scores were obtained on 345 children at T3. It was expected that SSEL would have both direct and indirect effects on kindergarten readiness through improvements in children’s SE and EF skills preschool academic skills and on-task behavior. We found no direct effects of SSEL on either pre-academic or on-task behavior outcomes in preschool, nor on later kindergarten readiness. However, SSEL significantly increased EF, and as expected by SSEL’s theory of change, growth in EF predicted gains in both pre-academics (particularly pre-math), and on-task behavior in preschool. End-of-year pre-academic skills and on task behavior in turn predicted better kindergarten readiness. Further, SE (although not impacted by SSEL) had direct and indirect effects on kindergarten readiness. Thus, overall, our findings largely support SSEL’s theory of change, particularly in relation to EF.

## Introduction

In recent decades there has been a heightened attention to addressing the achievement gap between disadvantaged children and their more affluent peers at school entry. For instance, national average math and reading scores of children in the highest SES group are more than a full standard deviation above the scores of the lowest SES group at kindergarten entry (García, 2015). Moreover, while the racial achievement gap has decreased over the last 50 years, the income achievement gap has widened as income inequality has grown (Reardon, 2013). In this regard, there has been a particular focus on better understanding the indicators and processes that contribute to school readiness (Blair and Raver, 2015), and the importance of intervening early to improve outcomes for disadvantaged children and narrow the achievement gap (Fox et al., 2010; Reardon, 2013). In addition to the development of acquired content skills (reading, math, oral language), research has focused on executive functioning (EF) and social-emotional skills as key elements linked to school readiness and academic success (La Paro and Pianta, 2000; Shields et al., 2001; Howse et al., 2003; McClelland et al., 2007; Bierman et al., 2008a; Denham et al., 2012; McClelland and Cameron, 2012; Baptista et al., 2016). Both EF (including working memory, inhibitory control, and attentional control/flexibility) and socio-emotional skills (e.g., emotion knowledge, perspective taking, social problem solving skills) assist children in the emotional, cognitive, and behavioral regulation needed to develop positive relationships with teachers and peers, and positive approaches to learning that are essential for accessing both formal and informal learning opportunities in the classroom, e.g., cooperating with others, managing stress, attending to and following directions, task persistence, etc. (Denham et al., 2012; Blair and Raver, 2015; Razza et al., 2015). In fact, kindergarten teachers report that social and regulatory behavior and attention skills are equally as important as content skills (e.g., pre-literacy, pre-numeracy) in setting the stage for academic success (West et al., 2001; Rimm-Kaufman et al., 2005). Unfortunately, many children, especially those from low-income families, enter kindergarten without these skills (Wanless et al., 2011; Raver, 2012; García, 2015).

### Executive Functioning

Executive functioning (EF) encompasses cognitive processes thought to support academic achievement through top down control of attention and behavior. While there is no consensus on the definition, EF is commonly referred to as an umbrella term for a set of cognitive control processes necessary for planning, problem solving, and monitoring behavior that is distinct from general intelligence (Blair et al., 2005; McClelland et al., 2007). EF includes working memory, or the ability to hold and manipulate information in memory, inhibitory control, or the ability to stop an automatic or preponderant response and/or substitute a subordinate response, and attentional flexibility, or the ability to regulate attention and shift attention as needed (Blair and Raver, 2015). Together, these skills in the beginning of the preschool year have been shown to positively predict literacy, vocabulary and math skills at the end of preschool (McClelland et al., 2007; Welsh et al., 2010), math and reading in kindergarten (Blair and Razza, 2007; Welsh et al., 2010), and math at age 7 (Bull et al., 2008), although some have found only concurrent, not predictive associations between EF and academic skills (Willoughby et al., 2012). EF has also been found to mediate the relationship between SES and academic achievement growth in both early and later childhood (Nesbitt et al., 2013; Lawson and Farah, 2017), suggesting its potential importance for efforts to address the achievement gap. Finally, poorer EF has been found to be related to behavior problems and ADHD (Willoughby et al., 2011; Schoemaker et al., 2012; Kim et al., 2013; Schoemaker et al., 2013).

### Social Emotional Skills

Social emotional skills are thought to be another important contributor to school readiness, although findings in this area are mixed and often show indirect but not direct relationships. In particular, research shows that SE skills such as emotion knowledge and social problem-solving skills are consistently related to social competence and fewer behavior problems (Smith, 2001; Denham et al., 2003; Denham et al., 2013), and sometimes directly related to academic competence (Garner and Waajid, 2008; Curby et al., 2015). Social emotional competence is important for school readiness in that it promotes adaptive learning behaviors such as cooperation, managing emotion and motivation on difficult tasks, and positive engagement with teachers and peers (Coolahan et al., 2000; Denham et al., 2014). Other research suggests EF (particularly attention) plays a mediating role between SE and academic outcomes (Trentacosta and Izard, 2007; Rhoades et al., 2011), while still others find SE mediates the effect of EF on academic outcomes (Walker and Henderson, 2012).

### Intervening to Improve Social Emotional and Executive Functioning for School Readiness

Early childhood is a key time in the development of these self-regulatory skills. Both EF and social emotional skills (SE) show rapid growth during the preschool years, mirroring the neurological growth and connectivity of the prefrontal and anterior cingulate cortices during this time (Bush et al., 2000; Blair and Razza, 2007). While these skills and processes begin in infancy and continue into later childhood and adolescence, showing increasing complexity and integration, there is a surge in growth between the ages of 3 and 7 (Riggs et al., 2006; Carlson and Wang, 2007). However, this rapid neurological growth and connectivity is heavily influenced by the environment—early experiences interact with individual characteristics of the child (e.g., temperament) to shape the development of EF and SE skills and the brain architecture that supports them (Blair, 2002). In particular, having stable, nurturing interactions with caregivers who provide modeling, scaffolding, and positive reinforcement, and home and school environments with predictable routines and opportunities for creative play and positive peer interaction help to build these self-regulatory skills. In contrast, environments characterized by excessive stress, neglect, or high levels of negative interactions impede the development of these skills (Center on the Developing Child at Harvard University, 2011; Blair and Raver, 2015; Bierman and Torres, 2016).

Because of the rapid developmental growth of SE and EF skills in early childhood, their sensitivity to malleable environmental conditions, and their importance to school readiness and success, the preschool classroom is one obvious setting to directly promote and support the development of these skills, particularly for disadvantaged children who may be at risk before they even begin formal schooling (Raver, 2012). As such, several classroom interventions and curricula designed to build social skills and self-regulation in preschoolers have been developed. Studies have shown they can be effective in improving low-income children’s social skills (Domitrovich et al., 2007; Morris et al., 2014), EF (Diamond et al., 2007; Bierman et al., 2008a,b; Raver et al., 2011; Upshur et al., 2017; Solomon et al., 2018), and sometimes pre-academic skills (Bierman et al., 2008a; Raver et al., 2011). Further, some evidence suggests that improvements in SE, and especially EF skills, mediate intervention effects on pre-academic outcomes (Bierman et al., 2008b; Raver et al., 2011; Ursache et al., 2012; Jones et al., 2013).

For instance, Raver et al. (2011) in an efficacy study of the Chicago School Readiness Project (CSRP) found EF mediated CSRPs effects on letter-naming skills and early math skills. Jones et al. (2013) using structural equation modeling (SEM), found that the effects of CSRP on children’s academic and behavioral outcomes were mediated by both teacher–child relationship quality and EF skills, that is, CSRP improved teacher-child relationships which in turn improved EF skills, which were, in turn, related to better math and reading as well as lower internalizing and externalizing behavior. In a study of the Head Start REDI project, Bierman et al. (2008b) found both moderation effects for EF (those children with lower baseline EF benefitted more) and mediating effects, whereby changes in EF partially mediated REDI’s effects on vocabulary and phonological sensitivity. However, more research is needed to elucidate the causal paths through which interventions may improve school readiness for at risk populations, and these must be driven by explicit hypotheses based on the intervention model. As Shonkoff (2017, p. 1) states, “When interventions are not linked to specific impacts based on explicit causal hypotheses, the variability of effects on multiple measures at different points in time is impossible to interpret and is rarely replicated.”

This study examines kindergarten readiness skills, independently assessed by schools, of one commercially available curriculum, Second Step Early Learning (SSEL, Committee for Children, 2011). SSEL is designed to impact SE and EF skills in preschool, with the ultimate goal of improving school readiness and kindergarten success. SSEL has shown some promising results but has not been widely studied. In a preliminary study using the first cohort of our study sample, we found children in SSEL classrooms showed greater preschool growth in EF, but only marginal growth in SE relative to controls (Upshur et al., 2017). In a follow up study using the whole sample, the effects for EF were maintained, but there were no significant effects for SE (Upshur et al., in review). We also found that SSEL classrooms had greater improvements in classroom quality (particularly emotional supportiveness), however, there were no direct effects on preschool academic outcomes and longer-term kindergarten outcomes were not reported (Upshur et al., in review). Using the curriculum’s logic model and developmental theory, the current study explores the direct and indirect pathways through which SSEL impacts kindergarten readiness.

### Second Step Early Learning Curriculum

The Second Step Early Learning Curriculum (Committee for Children, 2011) is a preschool curriculum targeted to 4–5-year old’s, but suitable for use in mixed age (3–5-year-old) classrooms. SSEL has 28 weekly themes divided into five units with daily large or small group activities to introduce and practice skills. It is guided by the extensive research base on self-regulation and social competence and their importance for school readiness. It is designed to build EF through both directly teaching EF skills (Unit 1: Skills for Learning, and Brain Builder games played daily), and through the use of instructional teaching methods that support children’s attentional skills, memory, and impulse control (using ‘think-time’ e.g., asking children to quietly think about an answer before raising their hand, and having the teacher wait 5 s before calling on children; using non-verbal cues like the ‘attentoscope,’ e.g., having children make a circle around their eyes with their hands like binoculars to help them focus their attention; reviewing the listening rules before starting each circle time; modeling and encouraging children to use ‘self-talk’ to help them remember). Social emotional skills are also promoted through directly teaching about emotions, emotion management strategies, and social problem-solving skills, as well as instructional teaching methods that foster child learning engagement (having children use non-verbal agreement, e.g., “If you agree with Jaydon, pat your head”; having children answer together, e.g., “How is the boy on the story card feeling?” all answer “sad”; calling on children at random to encourage those who need to be re-engaged or who may be shy), and strategies that promote positive behavior through emotional supportiveness, positive reinforcement and modeling strategies (e.g., focusing on and reinforcing positive behavior rather than negative behavior, modeling the calm-down strategies to manage emotions).

Although not scripted into the daily activity, teachers are also encouraged to reinforce skills throughout the day/week using strategies such as ‘think ahead’ (e.g., “We just learned about being kind and helpful. How might you be kind and helpful to your friends on the playground?”) and ‘think back’ (e.g., “I saw you both wanted that truck during free play. Which ‘fair ways to play’ did you use to solve the problem?”), as well as through creating practice opportunities in the classroom, noticing and reinforcing children when they use learned skills, and creating curriculum connections that extend and support newly learned skills (e.g., art projects that explore feelings; science projects that support problem-solving skills; developing a feelings bar chart to support numeracy skills). SSEL also includes a weekly handout for parents that teachers can copy and send home to provide information about what children are learning that week, with activities parents can do with their children at home to reinforce concepts.

The curriculum comes in a kit with a teacher manual, a boy and girl puppet, 28 large weekly theme cards with a full-color photo of diverse preschool children in situations relevant to the weekly theme (e.g., two children pulling on the same toy with angry faces) and instructions for each day’s activity on the back. Materials to support and reinforce skills throughout the week are also provided, such as a cd of theme-related songs, posters to hang in the classroom, and cards with feeling faces that can be used in matching games or for children to use to identify how they are feeling.

The underlying theory of change is that SSEL will lead to greater social emotional skills and EF, and that these skills will improve children’s on-task behavior and academic skills, which in turn will result in better kindergarten readiness.

### The Present Study

Our prior study examined the effects of SSEL on SE and EF and pre-academics but did not include kindergarten data. We found a significant impact of SSEL on EF skills but not on SE or pre-academic skills. The goal of the current study is to use SEM to examine SSEL’s effect on kindergarten readiness and the indirect and direct roles of end of preschool SE, EF, pre-academic skills and task behavior guided by the theory of change.

We expected that:

(1) Kindergarten readiness will be significantly higher for children from intervention classrooms compared to comparison classrooms.
(2) The effects of SSEL on kindergarten readiness will be mediated by end of year SE and EF skills, pre-math skills, pre-literacy/language skills, and on-task behavior.

## Materials and Methods

This study was approved by the Internal Review Board of the University of Massachusetts Medical School.

### Participants

Participants were 972 pre-kindergarten children recruited from 63 preschool classrooms within 13 childcare sites serving children aged 3 to 5 (55.2% were enrolled in Head Start classrooms; 44.8% in community childcare classrooms). Families were recruited in the fall of each year by human subjects research-trained childcare administrators, at the time of enrollment into the preschool program. Families who could not complete the informed consent process in English or Spanish were not recruited into the study. Only one child per family was maintained in the study. When more than one child from the same family was enrolled in the preschool, the oldest child enrolled was maintained, or a randomly selected child in the case of twins. Classrooms were frequently multi-aged (3–5 years), but only children aged 4–5 years or expected to enter kindergarten the next year were individually assessed by study staff.

### Procedure

Individual child assessment measures used in the current paper (all SE, EF, Pre-academic skills, and Task Behavior measures described in detail below) were collected in the fall (T1: collected between mid-September and mid-November; *n* = 972) and again in the spring [T2: collected between the end of March and the beginning of June of each year (2013 – 2017; *n* = 829 to 836, depending on the measure; see **Table 2**; mean age = 58.9 months)]. The study included two cohorts of preschool classrooms/teachers that were each involved in the study for 2 years (Cohort 1 in years 1 and 2; Cohort 2 in years 3 and 4 of the study). The present study includes both cohorts. For findings from Cohort 1, see Upshur et al. (2017). In addition, short-term preschool outcomes on the full sample also have been submitted for publication (Upshur et al., in review).

Children were assessed by trained child assessors (undergraduate or graduate students, or retired teachers) who were blind to study goals and conditions. Assessors were told only that it was a study of children’s development and learning. Debriefing of assessors after study involvement confirmed that none were aware that it was an intervention study. The assessment battery was completed during two, 30-min sessions on two different days (usually within the same week). Measures were assessed in the same order across children, with measures ordered to maximize engagement, attention and motivation, and minimize fatigue. Most assessments were conducted in a corner of the classroom because of preschool policies around child safety, but some sites allowed assessments to be conducted outside of the classroom in a common area.

Children enrolled in the first 3 years of the study were followed into kindergarten (*n* = 526) in 24 different school districts, private or charter schools. Kindergarten screening assessments (T3: collected at the beginning of the kindergarten year) using the Early Screening Inventory-Revised (ESI-R, Meisels et al., 2008) were available from school records for 342 (65%) of these children. The remainder of the sample followed into kindergarten either had various other screening measures that were not easily combined with ESI-R data or had no screening data reported.

#### Randomization

Preschool sites were randomly assigned to one of two cohorts. Each cohort was involved in the study for 2 years. Classrooms within each site were randomly assigned to condition at the beginning of the 1st year of their involvement (Years 1 or 3). Random assignment was conducted after children were enrolled and baseline data were collected. After random assignment, teachers in intervention classrooms were trained in the SSEL curriculum, with continued support over the 2 years of study involvement. In Years 2 and 4 new children entered previously randomized classrooms, while children also left the centers, so there was a combination of new and continuing students in many classrooms.

#### Teacher Training

Teachers were trained during 2-h evening meetings that provided information on the theoretical importance of the skills taught, reviewed implementation strategies, provided opportunities for practice, and most importantly, included time for teachers to share successes and trouble-shoot difficulties. There were 7 meetings in the 1st year of involvement (November–May) and 5 meetings (bimonthly from October–May) in the second year of involvement. In addition, project staff observed teachers implementing curriculum activities in their classrooms each month and provided written feedback. Fidelity was rated during these observations to monitor implementation (see Upshur et al., 2017 for more details about training and implementation support).

### Measures

#### Demographics

Parents provided demographic information including the parents’ marital status, education level, and family income, as well as the child’s sex, age, and ethnicity at the time of enrollment into the study.

#### Executive Functioning

The Head-Toes-Knees-Shoulders Task (HTKS, Ponitz et al., 2008) is a commonly used executive function task that taps all three aspects of EF: working memory, inhibitory control, and attentional flexibility. HTKS has three parts (each with 10 trials). The first part of the task requires children to touch their head when instructed to touch their toes (and vice versa). In the second part, in addition to the head and toes instruction, children must touch their knees when instructed to touch their shoulders (and vice versa). Finally, in the last part, the rules switch whereby children must touch their knees when told to touch their head (and vice versa) and touch their toes when instructed to touch their shoulders (and vice versa). Responses are scored as “0” if the child does not touch the correct body part, “1” if the child makes a move toward the wrong body part but then self corrects, and “2” if the child directly touches the correct body part. A total score is summed across the three parts (range 0–60; α = 0.84).

The Less Is More Task (LM; Carlson et al., 2005) was used to measure working memory and inhibitory control when an extrinsic reward is involved (called “hot” EF; Kim et al., 2013). Children are asked to choose between two plates, one plate having five stickers and the other having two stickers. Once it is established that the child would like the plate with more stickers, they are introduced to a “naughty monkey” named Chris (a stuffed animal) “who likes to get all the stickers for himself.” Children are then told that whatever plate they choose, the stickers in that plate will go into Chris’s bowl (Chris and his bowl are placed in front and to the right of the child). The stickers in the plate not chosen will go into the child’s bowl (placed in front and to the left of the child). Children are told that at the end, they can take all the stickers in their bowl home. Thus, children must inhibit a dominant response for choosing the plate with five stickers and instead, choose the plate with two stickers in order to get more stickers. In some trials, plates with 5 stickers were placed on the child’s right and sometimes on their left. Children were given 10 trials (with a rule reminder after 5). Children received 1 point for every trial in which they picked the plate with 2 stickers and a total score was the proportion correct across the 10 trials (range 0–100). Alpha reliability in this sample was adequate (α = 0.75).

The Backward Digit Span test (Davis and Pratt, 1996) was also included as a measure of working memory. Children were introduced to a puppet named Ernie who helped to demonstrate the correct response during the practice trials. During the demonstration, the examiner explained, “Whatever I say, Ernie says it backward. Like this, if I say “1, 2,” Ernie says “2, 1” (having the puppet respond). Now you try it. Whatever I say, you say it backward.” Children were given up to six practice trials prior to the test trials. The test trials start with two digits and progress to five digits with up to three attempts at each digit length. The highest string of numbers correctly repeated backward is scored (range = 1–5).

#### Social Emotional Skills

The Emotion Matching Task-Short Form (EMT-Short Form, Seidenfeld et al., 2014) was used to measure emotion knowledge. Children are shown photographs of racially diverse boys and girls with various facial expressions, including happiness, sadness, anger, fear/surprise and neutral. It has four parts, each with six items: (1) matching the emotional expression of a stimulus child to one of four other target children, e.g., “Show me which one of these children feels the same way as this one”; (2) matching emotions with situational cues, e.g., “Show me the one who just got a nice new toy”; (3) expression labeling, e.g., “Look at his face. How does he feel?”; and (4) matching pictures with emotional labels, e.g., “Show me the one who feels sad.” The scale correlates well with other widely used measures of emotion knowledge (Seidenfeld et al., 2014) and reliability for this sample was adequate (α = 0.82). In the current study, the total score was used (range = 0–30).

The Challenging Situations Task (CST, Denham et al., 1994) was used to assess children’s social problem solving. Children are shown pictures of hypothetical peer conflict situations. Three of the situations involve physical aggression (being hit, having a toy taken away) and three involve social aggression (e.g., being made fun of). For each situation, children are asked “What would you do if this happened to you?” Children are then presented with four pictures of behavioral responses (prosocial, aggressive, avoidant, or crying) given in random order across the six situational trials. Frequency counts for each behavioral response are calculated across the six situations. The total proportion of aggressive and prosocial responses were used for analyses (range = 0–1.0).

#### Preschool Academic Achievement

Pre-math, and pre-literacy/oral language skills were assessed at the beginning and again at the end of the preschool year using subtests of the Woodcock–Johnson Tests of Achievement III (WJ III, Woodcock et al., 2001/2007). Pre-math skills were measured using the WJ III Applied Problems subtest which measures counting and basic math skills. Pre-literacy/language skills were measured using three WJIII subtests: Letter-Word Identification, Understanding Directions, and Story Recall. Letter-Word Identification is a test of basic reading ability, requiring the child to identify letters and words. Understanding Directions is a test of oral language and listening comprehension, and requires the child to listen to examiner directions and then wait until the end of the directions when the examiner says “go” to point to various details in a picture in the correct order, e.g., “Point to the tiger, then the snake, and then the flower. Go.” Story Recall is a test of oral language, language development and meaningful memory requiring the child to listen to a story and repeat it back to the examiner. Points are totaled for number of correct story elements articulated. These tests are appropriate for age 4 and older, and measure key school readiness abilities (McGrew et al., 2007). The reported alpha reliability for these WJ III subtests ranged from a low of 0.77 for Story Recall to 0.99 for Letter-Word Identification (McGrew et al., 2007). Standard scores for each test were used in analyses.

#### Task Behavior

At the end of the individual child assessments, assessors rated the child’s task behavior using the Preschool Self-Regulation Assessment (PSRA) Assessor Report (Smith-Donald et al., 2007). This is a 28-item measure that was adapted from the Leiter-R social-emotional rating scale (examiner version; Roid and Miller, 1997) and the Disruptive Behavior-Diagnostic Observation Schedule coding system (DB-DOS; Wakschlag et al., 2005) as a global report of the child’s behavior during the assessment period. Items are rated on a 4-point scale. In this study, the Attentive/Impulse Control 2 subscale was used to measure task behavior, including 17 items that assess the child’s attention to instructions (e.g., “Pays attention during instructions and demonstrations,” “Careful, interested in accuracy; not careless”), behavior with materials (e.g., “Refrains from indiscriminately touching test materials,” “Let’s examiner finish before starting task; does not interrupt”), as well as the child’s cooperativeness, defiance, and emotional regulation (e.g., “Cooperates: complies with examiner’s requests,” “Defiant,” “Across the whole assessment period, modulates and regulates arousal level in self—keeps ‘an even keel”’). Negative items were reverse scored, and a mean score was calculated across the 17 items in the subscale, with a higher score indicating better attention and impulse control, and positive emotion (range = 0–3). Because assessments were conducted on two different days, assessors rated the child on day 1 and then on day 2, updated scores to reflect behavior across the 2 days. Alpha reliability for this scale in the present sample was 0.92.

#### Kindergarten Readiness

The Early Screening Inventory-Revised (ESI-R, Meisels et al., 2008) is a widely used developmental screening instrument designed to identify children who may be at risk for school failure and need extra assistance or special education services to succeed in kindergarten. The individually administered assessment tests children’s visual motor/adaptive skills (e.g., eye-hand coordination, ability to reproduce two and three-dimensional forms), language and cognition (language comprehension, verbal expression, counting), and gross motor skills (gross motor coordination, ability to imitate body positions from visual cues). It takes 15–20 min to administer, has a reported sensitivity of 92% and specificity of 80%, with good inter-rater (0.97–0.99), and test–retest (0.87–0.98) reliability. The total ESI score (which does not include any subscale scores) was obtained from school records and used in the present analysis.

### Data Analysis

The goals of the present analyses were to investigate the longitudinal effects of the SSEL curriculum on kindergarten readiness and to explore the mediating roles of SE, EF, academic skills and task behavior in preschool in predicting children’s kindergarten readiness guided by the curriculum’s ToC, while accounting for children’s nesting within classrooms. SEM was used to test the causal relationship among exposure to the SSEL curriculum, gains in preschool skills, and kindergarten readiness. To handle the multilevel-structured data, we used the TYPE = COMPLEX command in Mplus (version 8; Muthén and Muthén, 1998/2017), which provides robust standard errors to account for non-independence of observations.

The SEM process in the current study included two steps: validating the measurement model, and simultaneously fitting the cross-lagged and the structural equation models. In the first step, we evaluated the measurement model by conducting confirmatory factor analysis (CFA) to delineate the relationships among indicators and underlying latent variables. In the second step, structural equation models were tested using path analysis to determine the longitudinal associations among EF, SE, academic skills and task behavior in preschool, and later kindergarten readiness. We conducted a cross-lagged model for EF and SE between fall and spring in preschool to test the direction of effects between EF and SE and estimated the structural model for the longitudinal impact of the intervention on the kindergarten readiness at the same time. This process allows for concurrent estimation of longitudinal influences of one construct on another, and vice versa, while controlling for contemporaneous correlations between the constructs and the stability of each construct over time, as well as the relation to distal outcomes.

We fitted models using chi-square statistics, as well as the root mean square error of approximation (RMSEA), the comparative fit index (CFI), the Tucker–Lewis index (TLI), and the standardized root mean square residual (SRMR). The overall fit of each model was determined using RMSEA, CFI, TLI, and SRMR as the chi square test is sensitive to relatively large sample size. For the RMSEA and SRMR, values of less than 0.08, and ideally below 0.05, were used to indicate an adequate and reasonable fit to the data (Browne and Cudeck, 1993; MacCallum et al., 1996; Hu and Bentler, 1999). Values of 0.90 or greater, and ideally above 0.95, were used to indicate good model fit for the CFI and TLI (Hu and Bentler, 1999; Kline, 2005; Raykov and Marcoulides, 2006). All statistical analyses were conducted using IBM SPSS Statistics software (Version 24.0) and Mplus (version 8; Muthén and Muthén, 1998/2017).

## Results

### Missing Data

Missing data were handled by using full information maximum likelihood (FIML) to estimate all relevant models. The FIML parameter estimates result in less bias and are more appropriate than other methods (e.g., data deletion and imputation) under the condition of data missing completely at random (MCAR) or missing at random (MAR), (Schlomer et al., 2010). In the current sample, a considerable amount of missing data occurred due to the decreased response rate across the time points (15% missing in preschool spring and 65% missing in kindergarten follow-up). For this reason, to estimate whether data were at least MAR, we examined the patterns of missing data using Little’s MCAR test (Little, 1988). The null hypothesis for Little’s MCAR test is that the data are MCAR, indicating the data are assumed to be MCAR when the pattern of missing values does not depend on the data values. The results of Little’s test confirmed that the data were MCAR: χ^2^(34244) = 6298.789, *p* > 0.10. Data found to be MCAR provides assurance that the overall distributions of variables would remain the same with or without those missing participants, so the remaining participants are representative of the full sample, except for negligible random differences. Accordingly, we retained the entire sample in analyses to maximize statistical power.

### Sample Characteristics

**Table 1** describes the baseline characteristics for the 972 children and families. The children’s average age was 52.98 months (*SD* = 4.01), and 51.3% were male. Children were racially diverse (42.3% Anglo-American, 26.3% African–American, and 39.7% Hispanic-American). Parents’ average age was 31.32 years (*SD* = 7.91), and the majority of parents were unmarried (72.5%). Approximately half of parents (51.8%) completed more than high school. The majority of parents (74.3%) reported family incomes less than $30,000. *T*-tests or Chi-square analyses were used to compare differences between intervention and the control groups across the multiple demographic variables. A Bonferroni correction for Type I error (*p* < 0.005) indicated no significant group differences.

**Table 1:** Baseline characteristics of children and families (*N* = 972)^1^.

	Whole sample (*N* = 972)		Intervention (*N* = 501)		Control (*N* = 471)		*p*-value
	%	*n*	%	*n*	%	*n*	
Child age in months Mean (*SD*)	52.98 (4.01)	972	53.11 (3.95)	501	52.84 (4.07)	471	0.29
**Child sex**							0.50
Male	51.3	499	50.3	252	52.4	247	
Female	48.7	473	49.7	249	47.6	224	
Parent age in years Mean (*SD*)	31.32 (7.91)	467	31.24 (7.97)	492	31.40 (7.86)	467	0.75
Parents are married	27.5	267	28.7	144	26.1	123	0.41
**Parental education**							0.64
<High school	12.7	123	12.6	63	12.7	60	
High school	33.6	327	34.7	174	32.5	153	
>High school	51.8	504	50.9	255	52.9	149	
**Family income**							0.74
<$10,000	25.7	260	27.7	139	24.7	121	
$10,000–$19,999	26.7	260	26.5	133	27.0	127	
$20,000–$29,999	21.9	213	20.2	101	23.8	112	
$30,000–$39,999	10.1	98	9.0	45	11.3	53	
$40,000–$49,999	4.9	48	5.0	25	4.9	23	
$50,000+	6.7	65	8.2	41	5.1	24	
**Child ethnicity**							
Anglo-American	42.3	411	44.9	225	39.5	186	0.09
African–American	26.3	256	25.0	125	27.8	131	0.31
Hispanic-American	39.7	386	35.9	180	43.7	206	0.01
Asian–American	2.0	19	2.6	13	1.3	6	0.13
Other	2.9	28	2.6	13	3.2	15	0.58

^1^*For some variables, n is not equal to 972. This is due to missing data or to participants indicating that they belong in more than category (i.e., child ethnicity).*

### Descriptive Statistics

Basic descriptive statistics among SE, EF, and academic skills and task behavior measures in preschool fall and spring, as well as the ESI in kindergarten are shown in **Table 2**. In order to estimate the impact of the intervention on individual measures, effect sizes (ES) were calculated in the context of nested data in Mplus. Results revealed that ESs (ranging from 0.02 to 0.28) were small and mostly favoring intervention children except for Letter-Word in preschool fall and spring, which favored controls.

**Table 2:** Descriptive statistics for measures by intervention and control.

	Whole sample (*N* = 972)		Intervention (*N* = 501)		Control (*N* = 471)		Effect size (ES)
	n	*M* (*SD*)	*n*	*M* (*SD*)	*n*	*M* (*SD*)	
**Preschool fall**							
EMT	959	20.83 (5.10)	495	21.15 (4.98)	464	20.48 (5.21)	0.13
CST prosocial	965	2.31 (1.61)	498	2.37 (1.62)	467	2.25 (1.61)	0.08
CST aggressive	965	0.91 (1.30)	498	0.83 (1.27)	467	0.98 (1.33)	-0.12
Backward digit	964	1.13 (0.41)	499	1.13 (0.41)	465	1.12 (0.41)	0.02
HTKS	967	8.56 (13.09)	498	9.29 (13.65)	469	7.78 (12.43)	0.12
Less is more	954	0.58 (0.27)	492	0.60 (0.28)	462	0.56 (0.27)	0.15
Applied problems	964	100.53 (11.20)	497	100.83 (11.74)	467	100.22 (10.59)	0.06
Letter-word	966	95.34 (12.64)	497	95.15 (13.23)	469	95.54 (11.98)	-0.03
Story recall	968	86.30 (17.83)	500	86.97 (18.32)	468	85.59 (17.28)	0.08
Understanding directions	965	88.08 (17.75)	497	88.63 (17.64)	468	87.49 (17.86)	0.06
PSRA	956	2.59 (0.42)	493	2.59 (0.41)	463	2.58 (0.43)	0.03
**Preschool spring**							
EMT	829	24.15 (3.64)	426	24.36 (3.58)	403	23.93 (3.70)	0.12
CST prosocial	835	2.72 (1.68)	426	2.78 (1.69)	409	2.66 (1.67)	0.07
CST aggressive	835	0.81 (1.41)	426	0.72 (1.37)	409	0.91 (1.44)	-0.14
Backward digit	836	1.33 (0.62)	428	1.39 (0.66)	408	1.25 (0.56)	0.23
HTKS	834	17.58 (16.96)	426	19.88 (17.29)	408	15.17 (13.29)	0.28
Less is more	830	0.72 (0.27)	425	0.73 (0.26)	405	0.70 (0.28)	0.12
Applied problems	832	101.55 (11.59)	427	102.24 (12.08)	405	100.82 (11.01)	0.12
Letter-word	832	96.26 (13.02)	427	95.49 (13.70)	405	97.06 (12.23)	-0.12
Story recall	835	90.97 (20.48)	428	91.88 (20.77)	407	90.01 (20.15)	0.09
Understanding directions	832	87.84 (17.24)	427	89.36 (17.00)	405	86.23 (17.37)	0.18
PSRA	830	2.69 (0.34)	424	2.70 (0.33)	406	2.67 (0.35)	0.11
**Kindergarten follow-up**							
ESI	342	22.43 (8.28)	177	22.87 (9.49)	165	21.95 (6.75)	0.11

*EMT, Emotional Matching Task; PROS, Challenging Situations Task Prosocial; AGG, Challenging Situations Task Aggressive; BD, Backward Digit Span test; HTKS, Head-Toes-Knees-Shoulder Task; LM, Less is More Task; LW, WJIII Letter-Word Identification; AP, WJIII Applied Problems; SR, WJIII Story-Recall; UD, WJIII Understanding Directions*.

Correlations for all variables are shown in **Table 3**. Correlations between the same measure at the fall and spring time points in preschool (e.g., the correlation between fall and spring measurements of the Emotion Matching Task) were moderate to high (*r* = 0.33–0.76). Otherwise correlations across measures or between preschool and kindergarten were minimal or small except for one correlation within the CST data. For example, within each time point (fall or spring in preschool) correlations among measures ranged from 0.03 (CST-Prosocial and Less is More preschool fall) to -0.53 (CST-Prosocial and CST-Aggressive preschool spring). Correlations between preschool measures and kindergarten readiness ranged from 0.01 (Backward Digit preschool fall and kindergarten ESI) to 0.24 (Letter-Word fall preschool and kindergarten ESI). In addition, correlations among demographics and condition and preschool and kindergarten measures were also minimal to small, ranging from 0.00 (White and fall EMT; Hispanic and spring PSRA), to -0.23 (Hispanic and Applied Problems in the spring).

**Table 3:** Individual level correlations between key variables^1^.

	1	2	3	4	5	6	7	8	9	10	11	12	13	14	15	16	17	18	19	20	21	22	23	24	25	26	27	28
**Preschool fall**	
(1) EMT	-																											
(2) PROS	0.20	-																										
(3) AGG	-0.20	-0.51	-																									
(4) BD	0.16	0.08	-0.10	-																								
(5) HTKS	0.30	0.21	-0.18	0.39	-																							
(6) LM	0.22	0.03	-0.08	0.22	0.29	-																						
(7) LW	0.35	0.23	-0.22	0.31	0.46	0.28	-																					
(8) AP	0.14	0.11	-0.12	0.17	0.22	0.06	0.43	-																				
(9) SR	0.17	0.09	-0.05	0.10	0.24	0.21	0.23	0.13	-																			
(10) UD	0.22	0.13	-0.12	0.23	0.32	0.19	0.48	0.29	0.25	-																		
(11) PSRA	0.28	0.19	-0.22	0.12	0.24	0.10	0.23	0.17	0.08	0.17	-																	
**Preschool spring**	
(12) EMT	0.49	0.11	-0.11	0.06	0.16	0.10	0.23	0.15	0.10	0.15	0.18	-																
(13) PROS	0.17	0.34	-0.21	0.11	0.23	0.05	0.19	0.08	0.07	0.13	0.15	0.11	-															
(14) AGG	-0.14	-0.23	0.33	-0.03	-0.19	-0.07	-0.18	-0.07	-0.04	-0.14	-0.18	-0.13	-0.53	-														
(15) BD	0.23	0.21	-0.16	0.45	0.48	0.22	0.41	0.28	0.11	0.32	0.11	0.11	0.23	-0.12	-													
(16) HTKS	0.35	0.22	-0.22	0.30	0.58	0.31	0.47	0.26	0.21	0.34	0.28	0.21	0.22	-0.18	0.49	-												
(17) LM	0.27	0.10	-0.10	0.17	0.28	0.33	0.30	0.10	0.13	0.23	0.10	0.12	0.17	-0.10	0.25	0.36	-											
(18) LW	0.24	0.18	-0.17	0.27	0.35	0.18	0.63	0.45	0.18	0.40	0.22	0.19	0.16	-0.16	0.39	0.42	0.28	-										
(19) AP	0.11	0.11	-0.12	0.20	0.21	0.05	0.44	0.76	0.10	0.27	0.12	0.07	0.06	-0.05	0.29	0.25	0.07	0.49	-									
(20) SR	0.22	0.10	-0.09	0.12	0.24	0.21	0.31	0.14	0.43	0.23	0.12	0.15	0.15	-0.11	0.21	0.34	0.22	0.33	0.14	-								
(21) UD	0.22	0.13	-0.10	0.21	0.28	0.17	0.41	0.21	0.16	0.46	0.15	0.18	0.18	-0.13	0.33	0.43	0.27	0.48	0.23	0.32	-							
(22) PSRA	0.25	0.10	-0.13	0.13	0.20	0.07	.23	0.13	0.07	0.16	0.40	0.18	0.19	-0.21	0.16	0.26	0.13	0.22	0.10	0.08	0.17	-						
**Kindergarten follow-up**	
(23) ESI	0.19	0.15	-0.10	0.01	0.07	0.17	0.24	0.12	0.06	0.21	0.10	0.16	0.16	-0.16	0.19	0.21	0.12	0.21	0.18	0.11	0.21	0.20	-					
**Demographics**	
(24) Gender	0.08	0.00	-0.10	-0.01	0.04	-0.05	0.00	-0.01	0.05	-0.03	0.14	0.09	0.00	-0.10	0.02	0.04	-0.10	-0.01	0.02	0.02	-0.03	0.10	0.06	-				
(25) White	0.00	0.01	-0.02	0.09	0.13	0.10	0.18	-0.02	0.04	0.17	0.03	-0.06	0.12	-0.10	0.16	0.19	0.13	0.22	0.00	0.10	0.21	0.05	0.11	-0.07	-			
(26) Black	-0.02	0.03	0.03	-0.08	0.02	-0.04	-0.03	0.17	0.07	-0.06	-0.04	0.08	-0.08	0.13	-0.04	-0.03	-0.07	-0.04	0.21	0.03	-0.09	-0.11	-0.07	-0.02	-0.27	-		
(27) Hispanic	0.03	-0.07	0.09	-0.10	-0.17	-0.15	-0.12	-0.20	-0.09	-0.14	-0.01	0.02	-0.06	0.09	-0.13	-0.16	-0.08	-0.15	-0.23	-0.12	-0.11	0.00	-0.16	0.04	-0.18	-0.31	-	
(28) Intv	0.07	0.04	-0.06	0.01	0.06	0.08	0.03	-0.02	0.04	0.03	0.02	0.06	0.04	-0.07	0.12	0.14	0.06	0.06	-0.06	0.05	0.09	0.05	0.06	0.02	0.06	-0.03	-0.08	-

^1^*p-values are omitted due to their lack of validity in the context of individual level correlations with nested data. EMT, Emotional Matching Task; PROS, Challenging Situations Task Prosocial; AGG, Challenging Situations Task Aggressive; BD, Backward Digit Span test; HTKS, Head-Toes-Knees-Shoulder Task; LM, Less is More Task; LW, WJIII Letter-Word Identification; AP, WJIII Applied Problems; SR, WJIII Story-Recall; UD, WJIII Understanding Directions; Intv, Intervention*.

### Measurement Model

We conducted measurement models to test CFA for estimating the latent variables of EF, SE, and Pre-Literacy/Language (see **Table 4**).

**Table 4:** Standardized factor loadings for indicators with latent constructs in CFA.

Construct/indicator	Standardized factor loading (*SE*)	Standardized factor loading (*SE*)	Standardized factor loading (*SE*)
	Pre-math model	Pre-literacy/ Language model	Self-regulation model
**SE fall**			
EMT	0.319 (0.045)***	0.307 (0.044)***	0.329 (0.046)***
CST prosocial	0.702 (0.031)***	0.708 (0.032)***	0.685 (0.029)***
CST aggressive	-0.683 (0.026)***	-0.683 (0.026)***	-0.694 (0.026)***
**EF fall**			
Backward digit	0.495 (0.042)***	0.483 (0.042)***	0.458 (0.043)***
HTKS	0.766 (0.032)***	0.786 (0.036)***	0.835 (0.033)***
Less is more	0.406 (0.032)***	0.394 (0.033)***	0.371 (0.032)***
**SE spring**			
EMT	0.143 (0.042)**	0.139 (0.042)**	0.154 (0.042)***
CST prosocial	0.728 (0.046)***	0.766 (0.045)***	0.729 (0.042)***
CST aggressive	-0.700 (0.037)***	-0.694 (0.035)***	-0.726 (0.037)***
**EF spring**			
Backward digit	0.659 (0.026)***	0.643 (0.028)***	0.619 (0.033)***
HTKS	0.756 (0.028)***	0.780 (0.025)***	0.822 (0.031)***
Less is more	0.438 (0.030)***	0.430 (0.031)***	0.410 (0.031)***
**Pre-literacy/Language fall**			
Letter-word		0.400 (0.043)***	
Story recall		0.486 (0.044)***	
Understanding directions		0.634 (0.042)***	
**Pre-literacy/Language spring**			
Letter-word		0.361 (0.047)***	
Story recall		0.486 (0.044)***	
Understanding directions		0.634 (0.042)***	

*^∗∗^p < 0.01, ^∗∗∗^p < 0.001*.

#### Pre-math Model

The initial fit of the Pre-Math model was inadequate: χ^2^(80) = 524.465, *p* = 0.000; RMSEA = 0.076 (90% CI [0.070, 0.082]); CFI = 0.844; TLI = 0.766; SRMR = 0.068. Re-specification by correlating error terms for same indicators across the time points (BD, HTKS, and LM for EF from fall to spring; and EMT, CST prosocial, and CST aggressive for SE from fall to spring) produced a adequate fit to the data: χ^2^(74) = 232.913, *p* = 0.000; RMSEA = 0.047 (90% CI [0.040, 0.054]); CFI = 0.944; TLI = 0.909; SRMR = 0.061. Compared to the initial model, the fit of the re-specified model was significant: χ^2^Diff(6) = 291.55, *p* < 0.001. Therefore, the re-specified model was used as the final measurement model. In the Pre-math model, all factor loadings were statistically significant (*p* < 0.01).

#### Pre-literacy/Language Model

The test of the initial fit was inadequate: χ^2^(144) = 1188.020, *p* = 0.000; RMSEA = 0.086 (90% CI [0.082, 0.091]); CFI = 0.721; TLI = 0.632; SRMR = 0.086. The model was re-specified to improve the model fit by correlating error terms for same indicators across the time points (BD, HTKS, and LM for EF from fall to spring; and EMT, CST prosocial, and CST aggressive for SE from fall to spring; Letter-Word, Story recall, Understanding Directions for Language from fall to spring). Results showed an adequate fit to the data: χ^2^(135) = 326.697, *p* = 0.000; RMSEA = 0.038 (90% CI [0.033, 0.044]); CFI = 0.949; TLI = 0.928; SRMR = 0.056. Compared to the initial model, the fit of the re-specified model was significant: χ^2^Diff(9) = 861.323, *p* < 0.001. The re-specified model was used as the final measurement model. In the Pre-literacy/language model, all factor loadings were statistically significant (*p* < 0.01).

#### Task Behavior Model

The initial fit of the task behavior model was inadequate: χ^2^(80) = 558.490, *p* = 0.000; RMSEA = 0.078 (90% CI [0.072, 0.085]); CFI = 0.812; TLI = 0.718; SRMR = 0.064. The model was retested after the re-specification by correlating error terms for same indicators across the time points (BD, HTKS, and LM for EF from fall to spring; and EMT, CST prosocial, and CST aggressive for SE from fall to spring). The resulting model fits were satisfactory: χ^2^(74) = 250.673, *p* = 0.000; RMSEA = 0.050 (90% CI [0.043, 0.056]); CFI = 0.931; TLI = 0.888; SRMR = 0.059. Compared to the initial model, the fit of the re-specified model was significant: χ^2^Diff(6) = 307.817, *p* < 0.001. The re-specified model was used as the final measurement model. The task behavior model indicated that all factor loadings were statistically significant (*p* < 0.01).

### Structural Model

**FIGURE 1:**
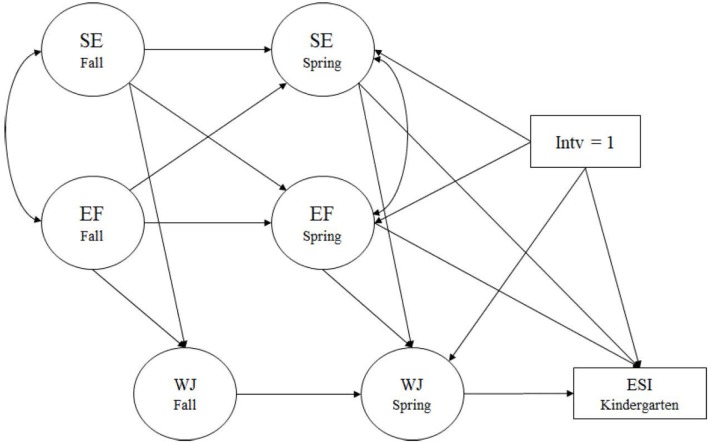
Hypothesized structural model. SE, social-emotional skills in preschool; EF, executive functioning in preschool; WJ, Woodcock Johnson III in preschool; Intv, intervention.

The structural model was conducted to explore the cross-lagged relationship between EF and SE and the intervention effect on kindergarten readiness through preschool EF/SE, early academic performance, and task behavior (see **Figure 1** for full hypothesized model). All hypothesized paths were included in our test of the full structural model, which included preschool EF, SE, and academic achievement as potential mediators of the relation between the SSEL intervention and the ESI kindergarten readiness scores. Next, the model was trimmed to eliminate non-significant paths. The final models show all statistically significant paths.

#### Pre-math Model

**FIGURE 2:**
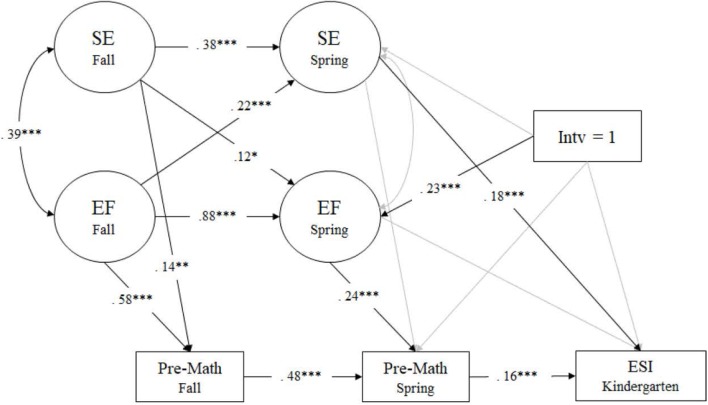
Math model measured by Applied Problems. Regression coefficients are standardized. *^∗^p* < 0.05, *^∗∗^p* < 0.01, ^∗∗∗^*p* < 0.001. SE, social-emotional skills; EF, executive functioning; Intv, intervention.

Results of the Pre-math model displayed an adequate fit to the data: χ^2^(90) = 252.855, *p* < 0.001; RMSEA = 0.043 (90% CI [0.037, 0.049]); CFI = 0.943; TLI = 0.924; SRMR = 0.064. In terms of the longitudinal model testing the relations between EF and SE across time, all paths were statistically significant except the correlation between EF and SE in spring. Both EF and SE were stable across time (β = 0.88 and 0.38, *p* < 0.001, respectively) and the synchronous correlation between EF and SE in fall was significant (*r* = 0.39, *p* < 0.001). The significant cross-lagged paths between EF and SE (β = 0.12, *p* < 0.05; β = 0.22, *p* < 0.001, respectively) allowed the inference of temporal precedence of EF to SE and vice versa. Preschool spring SE was directly related to ESI kindergarten (β = 0.18, *p* < 0.001) (see **Figure 2**).

Overall, the model showed that controlling for baseline, the intervention directly and positively influenced preschool spring EF (β = 0.23, *p* < 0.001), spring EF positively influenced spring Pre-math (β = 0.24, *p* < 0.001), and in turn, spring Pre-math positively predicted ESI in kindergarten (β = 0.16, *p* < 0.001). Model results showed the total effect and the direct effect were not significant (β = 0.07, *p* > 0.10; β = 0.06, *p* > 0.10, respectively). However, as Kenny and Judd (2014) acknowledge an indirect effect can still exist even when the total effect is not statistically significant. Accordingly, the indirect effect for mediation was validly tested (Loeys et al., 2015; O’Rourke and MacKinnon, 2015). Results for the indirect effect from intervention to ESI in kindergarten via preschool spring EF and Pre-math were significant (β = 0.009, *p* < 0.05), suggesting that the intervention was effective in promoting children’s kindergarten readiness indirectly via gains in EF and Pre-math skills in preschool.

To investigate the possibility that there was not enough statistical power to detect the total and direct effects of the intervention on ESI in this model, a simulation study was conducted in Mplus (version 8; Muthén and Muthén, 1998/2017) to assess the statistical power for those effects, following recommendations described in Muthén and Muthén (2002). Using the parameter estimates from our model as population values, 1000 data sets were generated with a sample size of 972 and the proportions of repetitions that produced statistically significant results for the total, direct, and indirect effects were used as estimates of statistical power. Based on this simulation, the significance test for the total effect had a statistical power estimate of 0.197, the significance test for the direct effect had a statistical power estimate of 0.165, and the significance test for the indirect effect had a statistical power estimate of 0.970. Thus, these results suggest that this study may have been underpowered to detect direct and total effects, but adequately powered to examine indirect effects.

Finally, given that both EF and Pre-math were assessed at the same time point and cannot be definitively causal in relation to one another, we further investigated whether the path from spring EF to spring Pre-math as hypothesized by the ToC, was justifiable (as opposed to spring Pre-math predicting spring EF). This was explored by comparing a series of cross-lagged models and examining the chi square difference between them to determine the best model fit. In each model, we correlated all fall variables (fall SE, fall EF, and fall Pre-math) and all spring variables (spring SE, EF, and Pre-math). Four models were compared. In the first baseline model, EF and SE in the spring were each predicted by their own and each other’s score from the fall, but spring Pre-math was only predicted by fall Pre-math. In the second model, the forward model, we replicated the first model but added paths from fall EF and fall SE to spring Pre-math. In the third model, the reverse model, we again replicated the first baseline model but instead of paths from fall EF and SE to spring Pre-math, paths were tested from fall Pre-math to spring EF and spring SE. The fourth model, the full model, had all fall variables predicting all spring variables. In this exploratory *post hoc* analysis, chi square analyses showed that the second model had the best fit, and the final significant paths in this model (as well as the full model) indicated that fall EF significantly predicted spring Pre-math. In fact, fall Pre-math did not significantly predict spring EF in either the reverse model or the full model. Taken together, these analyses suggest that while the direction of effects between spring EF and spring Pre-math cannot be definitively determined due to their concurrent assessment, the cross-lagged relation supports the direction of effects from EF to Pre-math.

#### Pre-literacy/Language Model

**FIGURE 3:**
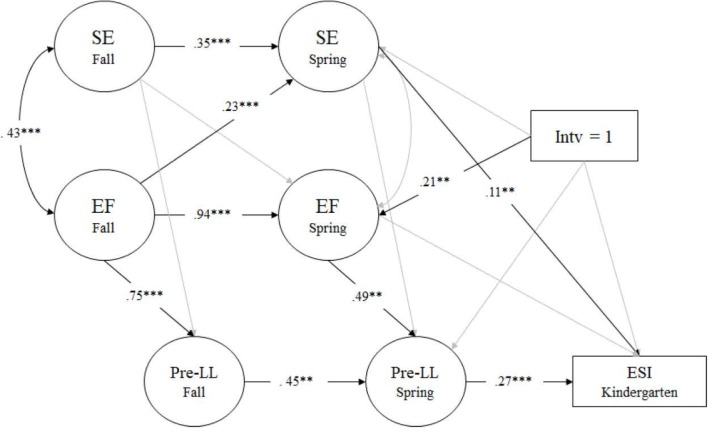
Pre-literacy/Language model measured by Letter-Word, Story Recall, and Understanding Directions. Regression coefficients are standardized. *^∗^p* < 0.05, *^∗∗^p* < 0.01, ^∗∗∗^*p* < 0.001. SE, social-emotional skills; EF, executive functioning; Pre-LL, Pre-literacy/Language; Intv, intervention.

Results of Pre-literacy/Language model exhibited an adequate fit to the data: χ^2^(153) = 367.241, *p* < 0.001; RMSEA = 0.038 (90% CI [0.033, 0.043]); CFI = 0.943; TLI = 0.929; SRMR = 0.059. In terms of the longitudinal association between EF and SE, we found that all paths were significant except the cross-lagged path from preschool fall SE to preschool spring EF and the correlation between preschool spring EF and SE. Both EF and SE were stable across time (β = 0.94 and 0.35, *p* < 0.001, respectively) and the synchronous correlation between EF and SE in preschool fall was significant (*r* = 0.43, *p* < 0.001). The significant cross-lagged path (preschool fall EF to spring SE, β = 0.23, *p* < 0.001) indicated that later SE was predicted by early EF. In addition, preschool spring SE directly predicted ESI kindergarten (β = 0.11, *p* < 0.01) (see **Figure 3**).

Again, we found that SSEL participation directly influenced preschool spring EF (β = 0.21, *p* < 0.01), with children in the intervention group showing higher levels of preschool spring EF than those in the control group. Further, preschool spring EF promoted spring Pre-literacy/Language (β = 0.49, *p* < 0.01), which in turn, positively influenced ESI in kindergarten (β = 0.27, *p* < 0.001). In this model, the total effect and the direct effect were not significant (β = 0.07, *p* > 0.10; β = 0.04, *p* > 0.10, respectively), while the indirect effect from intervention to ESI kindergarten via preschool spring EF and Pre-literacy/Language was significant (β = 0.03, *p* < 0.05). The results showed that the intervention influenced children’s kindergarten readiness indirectly through improvements in EF and Pre-literacy/Language in preschool.

As with the Pre-math model, we again investigated the possibility that there was not enough statistical power to detect the total and direct effects of the intervention on ESI in this model using a simulation study conducted in Mplus (version 8; Muthén and Muthén, 1998/2017). Based on this simulation, the significance test for the total effect had a statistical power estimate of 0.211, the significance test for the direct effect had a statistical power estimate of 0.115, and the significance test for the indirect effect had a statistical power estimate of 0.951, indicating possible inadequate power to detect total and direct effects, but adequate power to assess indirect effects.

Again, due to the concurrence in measurement, we further investigated whether the path from spring EF to spring Pre-literacy/Language was valid. As described above with the Pre-math model, we compared the four models using the Pre-literacy/Language outcome. In this analysis, model 1 had the best model fit. In model 1, the baseline model, all fall measures were correlated, all spring measures were correlated, EF and SE predicted themselves and each other over time, but spring Pre-literacy/Language was only predicted by fall Pre-literacy/Language. Neither of the cross-lagged models with EF predicting Pre-literacy/Language (models 2 and 4) nor the models with Pre-literacy Language predicting EF (models 3 and 4) fit the data as well as the simpler model where fall Pre-literacy Language predicted spring Pre-literacy/Language. This suggest that when it comes to Pre-literacy/Language, the causal path from spring EF to spring Pre-literacy/Language may not be valid. Although EF and Pre-literacy/language are correlated in the spring, we cannot from this follow up analysis, have confidence that gains in EF led to gains in Pre-literacy/Language. Therefore, the indirect effect of SSEL on kindergarten readiness through EF and Pre-literacy/Language may also not be justified.

#### Task Behavior Model

The test of the Task Behavior model displayed satisfactory fit to the data: χ^2^(89) = 261.331, *p* < 0.001; RMSEA = 0.045 (90% CI [0.038, 0.051]); CFI = 0.932; TLI = 0.909; SRMR = 0.063. With regard to the path model of the longitudinal relationship between EF and SE across time, all paths were statistically significant except the correlation between preschool spring EF and SE. Both EF and SE were stable across time (β = 0.87 and 0.40, *p* < 0.001, respectively) and the synchronous correlation between preschool fall EF and SE was significant (*r* = 0.37, *p* < 0.001). The significant cross-lagged paths between EF and SE (β = 0.20, *p* < 0.001; β = 0.12, *p* < 0.01, respectively) allowed the interpretation of reciprocal association from early EF to later SE and vice versa. A path between preschool spring SE and ESI kindergarten was also significant in this model (β = 0.19, *p* < 0.001).

**FIGURE 4:**
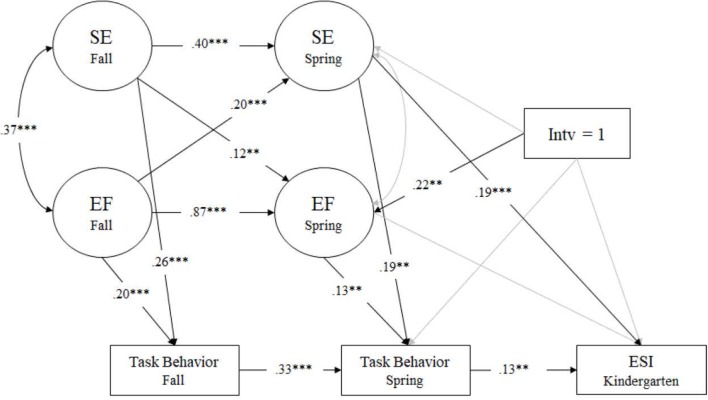
Task behavior model measured by PSRA. Regression coefficients are standardized. *^∗^p* < 0.05, *^∗∗^p <* 0.01, ^∗∗∗^*p* < 0.001. SE, social-emotional skills; EF, executive functioning; Intv, intervention.

As with the previous models, SSEL directly and positively influenced preschool spring EF (β = 0.22, *p* < 0.01), spring EF improved spring task behavior (β = 0.13, *p* < 0.01), and in turn, spring task behavior positively predicted ESI in kindergarten (β = 0.13, *p* < 0.01). Model results showed no significant total and direct effects (β = 0.08, *p* > 0.10; β = 0.08, *p* > 0.10, respectively), however, the indirect effect from intervention to ESI kindergarten via spring EF and spring task behavior in preschool was marginally significant (β = 0.004, *p* = 0.055) (see **Figure 4**).

As in the previous models, a simulation study in Mplus (version 8; Muthén and Muthén, 1998/2017) was again used to investigate the possibility that there was not enough statistical power to detect the total and direct effects of the intervention on ESI in this model, following recommendations described in Muthén and Muthén (2002). Based on this simulation, the significance test for the total effect had a statistical power estimate of 0.251, the significance test for the direct effect had a statistical power estimate of 0.237, and the significance test for the indirect effect had a statistical power estimate of 0.629. Lower statistical power for the task behavior indirect effect may explain the marginally significant relation between spring EF and task behavior and kindergarten readiness. As with the other models, a larger sample size may have resulted in stronger findings.

Finally, we investigated whether the path from spring EF to spring Task Behavior as hypothesized by the ToC, was justified. This was explored as above by comparing the four cross-lagged models (baseline, forward, reverse and full) and examining the chi square difference between them to determine the best model fit. Chi square analyses showed that the second model (the forward model) had the best fit, and the final significant paths in this model (as well as the full model) indicated that fall EF significantly predicted spring Task Behavior. In fact, fall Task Behavior did not significantly predict spring EF in either the reverse model or the full model. Taken together, these analyses suggest that while the direction of effects between spring EF and spring Task Behavior cannot be definitively determined due to their concurrent assessment, the cross-lagged relation supports the direction of effects from EF to Task Behavior.

## Discussion

This study examined the direct and indirect effects of the SSEL curriculum on kindergarten readiness guided by the curriculum’s theory of change (ToC). As of 2016, SSEL was being used by an estimated 7,900 preschool classrooms each year (Committee for Children, personal communication 4/17/2018). While widely used and grounded in a strong theoretical and empirical base, to date, our study has been the only published randomized trial of its effectiveness (Upshur et al., 2017; Upshur et al., in review). Further, the current study is the first to look at outcomes beyond preschool and explore the hypothesized paths through which the curriculum is expected to improve kindergarten readiness. Specifically, according to the curriculum’s ToC, it was expected that SSEL would improve children’s SE and EF skills, and that these skills would lead to better preschool academic skills and task behavior, which in turn would result in greater kindergarten readiness.

As in our previous studies (Upshur et al., 2017; Upshur et al., in review), we found SSEL significantly increased EF, but not SE. When the theoretical paths expected by the ToC were tested, the current study found end-of-year EF predicted end-of-year academic outcomes (pre-math and pre-literacy/language) and task behavior, controlling for baseline skills. The concurrent assessment of these measures in the spring, however, makes it impossible to test determinant or causal paths. While not conclusive, our follow up exploratory cross-lagged models do support this directionality, at least for Pre-math and Task Behavior. The fact that follow up cross-lagged models did not support the predicted path from EF to Pre-literacy/Language, but rather a concurrent association between the two, suggests that perhaps some third, unmeasured variable may be driving growth in both areas (e.g., teacher–child relationships or home environment). However, the literature in relation to EF and academics is stronger in relation to math than to literacy/language skills (Blair and Razza, 2007; McClelland et al., 2014; Schmitt et al., 2017), and our findings may reflect that. Our models also found, as expected, that pre-academic skills and on-task behavior in turn, predicted better kindergarten readiness. Previous research strongly links EF skills with academic development in preschool and kindergarten (Blair and Razza, 2007; McClelland et al., 2007; Welsh et al., 2010), and both EF and prior academic achievement have been shown to be the best predictors of later academic achievement (Duncan et al., 2007). While SSEL did not directly impact academic outcomes and task related behavior in preschool, it did increase the attentional, memory, and inhibitory control skills that supported academic learning, particularly for pre-math and on task behavior in preschool, and ultimately, school readiness at the beginning of kindergarten. SE had both direct and indirect effects on kindergarten readiness (through on task behavior gains, and early pre-math skills). Thus, overall, our findings largely support SSEL’s theory of change and previous research, at least in relation to EF.

Our finding that SSEL did not significantly increase SE compared to controls is congruent with our previously reported outcomes for SSEL using the full sample and may be explained by the fact that the control classrooms (particularly in the second cohort) were making a concerted effort to focus on SE skills, based on newly passed state regulations (Upshur et al., in review). SSEL had a marginal impact on SE in the first cohort (Upshur et al., 2017), and perhaps if conducted in classrooms not already emphasizing these skills, results may have significantly favored SSEL classrooms. Upshur et al. (in review) also reported that observations of teacher instruction across a subsample of both intervention and control classrooms confirmed that control classrooms were teaching similar levels of socio-emotional skills (although less emotion management), but intervention classrooms had greater instruction and support of EF skills. As such, more research is needed in broader geographic areas to determine SSEL’s relative effectiveness with regard to SE.

While we also did not find that SE was directly related to academic growth in preschool, it was directly related to gains in on task behavior, and had direct effects on kindergarten readiness across all three preschool models (Pre-math, Pre-literacy/Language and Task Behavior), emphasizing its critical importance in preschool education. The literature base is mixed in regards to SE’s effects on academic outcomes, with some finding direct effects (Garner and Waajid, 2008; Curby et al., 2015), some finding no effects (Duncan et al., 2007), and still others finding indirect relations (Walker and Henderson, 2012). The results of this study provide further evidence that, in the case of preschool academic outcomes, SE does not directly impact academic growth. However, at least for pre-math, the cross-lagged model showed that SE played an indirect role, both through pre-math skills at the beginning of the year (leading to better end-of-year pre-math) and through end-of-year EF, which predicted end-of-year pre-math, leading to kindergarten readiness. The latter finding is consistent with research that shows that EF mediates the relationship between SE and academic outcomes (Trentacosta and Izard, 2007; Rhoades et al., 2011) and highlights the importance of SE along with academics for successful kindergarten transition (Blankson et al., 2017). SE also had direct effects (along with EF) on task behavior, which predicted kindergarten readiness. Our measure of task behavior measured the child’s ability to cooperate, manage stress during difficult and sometimes repetitive tasks, follow directions and deal with distractions over the course of the two 30-min assessment periods (which were conducted in the classroom while other children were engaged in other classroom activities because of preschool safety rules). Thus, children’s positive task behavior during the testing situation required both top-down cognitive and attentional control (EF) as well as bottom-up emotional and behavioral (SE) self-regulation in order to both engage with the assessment tasks, as well as screen out the busy classroom environment. Similar measures of task behavior and approaches to learning (as reported by teachers) also show relations to EF and SE, and have been shown to be important for kindergarten readiness and school success (Kena et al., 2015; Razza et al., 2015; Vitiello and Greenfield, 2017).

Finally, we expected SSEL would have direct effects on kindergarten readiness, along with those mediated by EF and SE. However, we found no direct effects. This may be explained by low power in our study as our follow up simulation tests indicated. However, SSEL does not focus directly on academics or the types of skills measured in most kindergarten screening tests (e.g., language, counting, gross and fine motor skills). Further, our results were congruent with the results of other similar curricula. For instance, in a large randomized trial in Head Start classrooms comparing The Incredible Years, Preschool PATHS, and Tools of the Mind curricula to control classrooms, none of the interventions had direct effects on individually assessed preschool academic skills or teacher-rated kindergarten outcomes (Morris et al., 2014). In fact, similar to our findings, they found that two out of the three interventions had slightly lower end of preschool pre-reading and vocabulary scores than controls, and slightly lower kindergarten teacher-rated language and literacy scores. Their findings and ours underscore the need for including rather than supplanting academic instruction along with instruction to build SE and EF skills. In our study, the highest correlation with kindergarten readiness was with fall Letter-Word Identification (*r* = 0.24) indicating the importance of basic reading skills for kindergarten readiness, and the need to promote pre-literacy/language skills in preschool. It is possible that teachers in the intervention classrooms may have had less of an emphasis on pre-reading or pre-math skills than control classrooms who were not asked to implement a new curriculum. While SSEL includes reading a book about the weekly theme at the end of the week and encourages teachers to link SSEL learning with other curriculum goals (art, science, math, and literacy), it is not designed to directly build pre-reading or pre-math skills. SSEL is intended to be integrated into program goals and support curriculum standards [e.g., Head Start performance standards, or Teaching Strategies GOLD (Teaching Strategies LLC, 2018)^1^]. As such, other skills building such as literacy and numeracy also need to be continued. However, while further study of the long-term school impacts of SSEL is warranted, it appears all too common that gains from interventions by at-risk preschoolers fade out in later schooling, perhaps because of what kindergarten and elementary school teachers emphasize, or because of entrance into poor quality classrooms overall (Duncan and Magnuson, 2013).

This study has a number of strengths. It includes a large, diverse sample of low-income children in both Head Start and community preschools. It also relies on individual child assessments by staff blind to study goals and condition and follows children into kindergarten, obtaining kindergarten readiness scores from school records. However, the study also has several limitations. First, as mentioned previously, the design of the study (with SE, EF, pre-academic outcomes and task behavior assessed concurrently in the fall and spring of preschool), does not allow us to draw conclusions regarding direction of effects between the measures within the same time point. The overall study was not initially designed to test the ToC, but rather to test the efficacy of the curriculum to improve preschool and kindergarten outcomes, and was not funded to enable individual testing of children beyond preschool. However, other similarly designed studies have tested and reported mediational models with concurrently assessed measures (e.g., Raver et al., 2011), and our follow-up cross-lagged analysis provides some support for hypothesized paths, at least for Pre-math and Task Behavior. More research is needed, however, as a recent meta-analysis of school-based interventions targeting EF to improve academic achievement showed no evidence of a causal association (Jacob and Parkinson, 2015).

A second limitation is sample size at the kindergarten follow up. Only children entering the study during the first 3 years were followed into kindergarten, and there were a sizeable number of children for whom we were not able to obtain school kindergarten screening data. It is possible that a larger sample at the kindergarten time point may have improved power; however, it is unlikely that it would have changed the direction of effects or mediational paths, since data were shown to be MCAR. While a larger sample and increased power may have resulted in direct effects on pre-academic and kindergarten readiness outcomes, it is also possible that, similar to other intervention studies (e.g., Morris et al., 2014), SSEL does not directly affect preschool academic outcomes and kindergarten readiness (particularly given the heavy focus on pre-literacy and pre-numeracy that these screening assessments entail), but rather that the effects are more complex. As noted above, building EF and SE is important to support children’s learning behaviors (cooperation, following directions, emotional, and behavioral regulation) and may be a critical factor in improving academic growth and kindergarten readiness as suggested by our strong indirect effects for EF and direct effects for SE. However, while critical or necessary, improving EF and SE may not be enough. That is, without developmentally appropriate, high quality instruction, and language, literacy, and numeracy enriched learning environments, children (especially from low income, less enriched home environments) may not increase their academic skills and kindergarten readiness through improved EF and SE alone. Our strong indirect, yet weak direct effects suggests that these relationships may be more complex and that numerous other factors (home environments, teacher–child relationships, classroom structure and climate, child’s genetics, temperament, or experience of toxic stress) contribute more directly to kindergarten readiness than the curriculum itself. For instance, Bierman and colleagues report that adding home visits to their REDI classroom intervention for disadvantaged children (teaching parents to support their child’s socio-emotional and learning behaviors) had improved results over the preschool classroom curriculum alone on children’s academic functioning in both kindergarten (Bierman et al., 2015) and into second grade (Bierman et al., 2017). Perhaps with a larger sample size, direct effects would have been found for SSEL, but perhaps additional home environment interventions are also needed.

A third limitation of this study is that randomization of classrooms occurred in years 1 and 3 (the 1st year of each cohort), but in year 2, program administrators placed children in classrooms as needed, which could have introduced bias (e.g., children with more behavioral problems could have been placed in intervention classrooms) possibly biasing effects downward. Finally, since the task behavior ratings were based on the child’s behavior during the assessment of the other skills, rather than on teacher report, the relation between task behavior and individually assessed SE and EF may be inflated.

Overall, SSEL appears to have the expected effect on EF and the expected paths to kindergarten readiness are supported by this study. Future research with broader populations, and attention to not only the implementation of SSEL, but to other curriculum or instruction (e.g., pre-reading, pre-math), as well as following children beyond kindergarten entry is needed to fully understand the short and long-term impact that this curriculum has for children at socio-economic risk. However, SSEL appears to hold promise for improving school readiness and addressing the achievement gap for disadvantaged children by increasing EF with resulting improvements in pre-academic skills (particularly pre-math) and on-task behavior, leading to school readiness.

## Ethics Statement

This study was carried out in accordance with the recommendations of ‘the Human Research Protection Program, Internal Review Board of the University of Massachusetts Medical School.’ The protocol was approved by the Internal Review Board of the University of Massachusetts Medical School.

## Author Contributions

CU and MW-G conceptualized and implemented the study. MW-G wrote the introduction, methods and discussion sections of the manuscript. YY contributed to the coaching of teachers, conducted the data analyses, and wrote the data analyses and results sections. CU assisted in conceptualization of the manuscript and methods, and provided detailed editing of the full manuscript. AG provided significant guidance on the statistical analyses, conducted and reported the results of the follow-up Monte Carlo simulation tests, and assisted with the interpretation and write up of the results.

## Conflict of Interest Statement

The authors declare that the research was conducted in the absence of any commercial or financial relationships that could be construed as a potential conflict of interest.

## Footnotes

1 https://teachingstrategies.com/our-approach/our-38-objectives/
